# ^99m^Tc-A1 as a Novel Imaging Agent Targeting Mesothelin-Expressing Pancreatic Ductal Adenocarcinoma

**DOI:** 10.3390/cancers11101531

**Published:** 2019-10-10

**Authors:** Christopher Montemagno, Shamir Cassim, Dimitry Trichanh, Clara Savary, Jacques Pouyssegur, Gilles Pagès, Daniel Fagret, Alexis Broisat, Catherine Ghezzi

**Affiliations:** 1 LRB, CHU Grenoble Alpes, INSERM U1039, Université Grenoble Alpes, 38000 Grenoble, France; montemagno.chris@gmail.com (C.M.); dim.trichanh@gmail.com (D.T.); DFagret@chu-grenoble.fr (D.F.); catherine.ghezzi@univ-grenoble-alpes.fr (C.G.); 2 Biomedical Department, Centre Scientifique de Monaco, 98000 Monaco, Monaco; shamir_cassim@yahoo.fr (S.C.); Jacques.POUYSSEGUR@unice.fr (J.P.); Gilles.Pages@unice.fr (G.P.); 3 IGDR (Institut de génétique et développement de Rennes), UMR CNRS 6290, Université de Rennes, 35000 Rennes, France; clara.savary@univ-rennes1.fr; 4 Institute for Research on Cancer and Aging of Nice, Centre Antoine Lacassagne, CNRS UMR 7284, INSERM U1081, Université Côte d’Azur, 06200 Nice, France

**Keywords:** PDAC, Mesothelin, noninvasive imaging

## Abstract

Mesothelin is a membrane-associated protein overexpressed in pancreatic ductal adenocarcinoma (PDAC). Some mesothelin-targeted therapies are in clinical development but the identification of patients eligible for such therapies is still challenging. The objective of this study was to perform the imaging of mesothelin in mice models of PDAC with a technetium-labeled anti-mesothelin single-domain antibody (^99m^Tc-A1). Methods: The Cancer Genomic Atlas (TCGA) database was used to determine the prognostic role of mesothelin in PDAC. ^99m^Tc-A1 was evaluated both in vitro in PDAC cells (SW1990 and AsPC-1) and in vivo in an experimental model of mesothelin-expressing PDAC (AsPC-1) in mice. Results: TCGA analysis showed that PDAC patients with high mesothelin expression had a shorter overall survival (P = 0.00066). The binding of ^99m^Tc-A1 was 2.1-fold greater in high-mesothelin-expressing AsPC-1 cells when compared to moderate-mesothelin-expressing SW1990 cells (*p* < 0.05). In vivo, the ^99m^Tc-A1 uptake was 3.5-fold higher in AsPC-1-derived tumors as compared to a technetium-labeled irrelevant antibody (^99m^Tc-Ctl) (*p* < 0.01). Conclusions: ^99m^Tc-A1 accurately allows imaging of mesothelin-expressing experimental PDAC tumors. Our experiments paved the way for the development of a companion test for mesothelin-targeted therapies.

## 1. Introduction

Pancreatic ductal adenocarcinoma (PDAC) is one of the most aggressive tumors, representing the fourth leading cause of cancer-related deaths worldwide in 2018 [1]. By the year 2030, PDAC is projected to surpass breast, colorectal, and prostate cancer and to become the second most deadly malignancy [2]. Despite intense clinical research, the five-year survival rate remains just around 5–7% and one-year survival is achieved in less than 20% of cases [3]. Moreover, resistance to chemotherapy and lack of effective therapies contribute to the pejorative prognosis [4]. Finally, the majority of PDAC is diagnosed at advanced stages, thereby limiting therapeutic windows to manage patients [5].

The mesothelin gene encodes a 71 kDa precursor protein. It is processed in a shed form known as Megakaryocyte Potentiating Factor, and a 40 kDa GPI-anchored membrane form, which is the mesothelin protein itself [6]. Mesothelin expression is limited to mesothelial cells of the pleura, peritoneum, and pericardium. In normal tissues, the expression of mesothelin is very weak. However, its overexpression has been evidenced in several types of cancers including ovarian cancers and mesothelioma. Hence, mesothelin has been identified as a diagnostic marker and a relevant therapeutic target. As much as 80–85% of PDAC displayed greater levels of mesothelin [6,7]. No significant elevation of the shed form was detected in the serum of PDAC patients. The major form in cancers is membrane-associated mesothelin [8]. Mesothelin-targeted therapies are currently on clinical evaluation for the management of PDAC patients. However, the identification of patients eligible for such therapies still remains a challenging issue [6].

We recently validated ^99m^Tc-A1 as a single-domain-based imaging agent used for the phenotypic imaging of membrane mesothelin-expressing breast cancer [9]. In this study, we tested the ability of ^99m^Tc-A1 in imaging mesothelin-expressing PDAC tumors.

## 2. Results

### 2.1. Overexpression of Mesothelin in PDAC Patients Is Associated with Poor Clinical Outcomes

**Figure 1:**
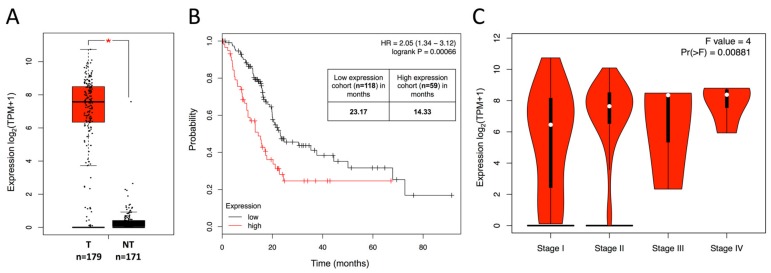
Prognostic value of mesothelin expression by pancreatic ductal adenocarcinoma (PDAC) patients for survival. (**A**) Expression of mesothelin in tumoral (T) and nontumoral (NT) pancreatic tissues from The Cancer Genomic Atlas (TCGA) and Genomic Tissue-Expression (GTEx) datasets. The red and gray boxes represent PDAC and nontumoral-derived tissues, respectively (T: n = 179 and NT: n = 171). (**B**) Kaplan–Meier plots of overall survival probability (plotted on Y-axis) of PDAC cancer patients is shown (TCGA data, n = 177). Patients have been stratified into high (red lines, n = 59) or low (black lines, n = 118) expression-based “risk-groups” by their gene expression of mesothelin. The patient follow-up is indicated in months on the X-axis. Respective log-rank test *p*-value, HR, and computed median survivals of low and high expression cohorts in months are shown and were calculated at the best auto-selected cut-off. (**C**) Violin plot showing the average gene expression levels of mesothelin at early (I and II) and advanced (III and IV) cancer stages of PDAC patients (TCGA database, n = 179). * *p* < 0.05.

Tumoral PDAC-derived specimens demonstrated significant increased levels of mesothelin (*MSLN*) when compared to peritumoral (nontumoral) pancreatic tissues (Figure 1A) (n = 179 and 171, respectively; *p* < 0.05). PDAC patients with high *MSLN* tumoral gene expression had a significant decreased overall survival when compared to patients with low expression (Figure 1B) (n = 177; P = 0.00066; HR: 2.05). Moreover, an elevated expression pattern was only observed in advanced stages (comparison of stages I and II to stages III and IV, *Pr*(>F) = 0.00881) (Figure 1C). To further validate our PDAC in silico dataset study model, greater expression of proliferative markers *MKI67*, *CCNB1*, and *PCNA* were only depicted in tumoral PDAC-derived specimens (Figure S1A, *p* < 0.05) and their overexpressions were associated with a shorter overall survival (Figure S1B, *p* < 0.01).

### 2.2. ^99m^Tc-A1 Binding on Mesothelin-Expressing PDAC Cell Lines

**Figure 2:**
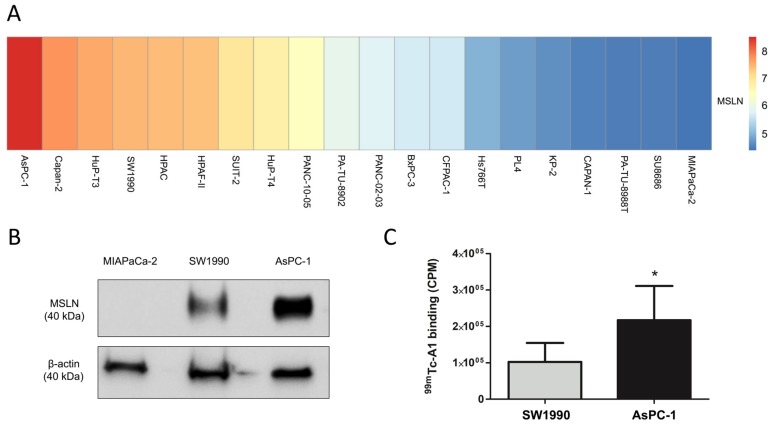
^99m^Tc-A1 binds to mesothelin-expressing cells in vitro. (**A**) Heatmap displaying *MSLN* gene expression levels across 20 PDAC cell lines. (**B**) Mesothelin expression of MIAPaCa-2, SW1990, AsPC-1 cells was assessed by Western blot. (**C**) Binding of ^99m^Tc-A1 to SW1990 and AsPC-1 cells (n = 6 per condition). Results were expressed in counts per minute (CPM). * *p* < 0.05 vs. SW1990.

Through an unbiased in silico approach, mesothelin expression was assessed in 20 PDAC cell lines. An increased, moderated, and reduced mRNA expression of mesothelin was evidenced in AsPC-1, SW1990, and MIAPaCa-2, respectively (Figure 2A). Based on this observation, high-, medium-, and low-MSLN-expressing PDAC cell lines were selected for in vitro characterization. Mesothelin protein was expressed by AsPC-1 and SW1990 but not by MIAPaCa-2 cells (Figure 2B, Figure S2). ^99m^Tc-A1 binding was then assessed on these cell lines (Figure 2C). ^99m^Tc-A1 binding was 2.1-fold higher in AsPC-1 as compared to SW1990 cells (*p* < 0.05).

### 2.3. SPECT-CT Imaging of Mesothelin in Subcutaneous Tumor Model

**Figure 3:**
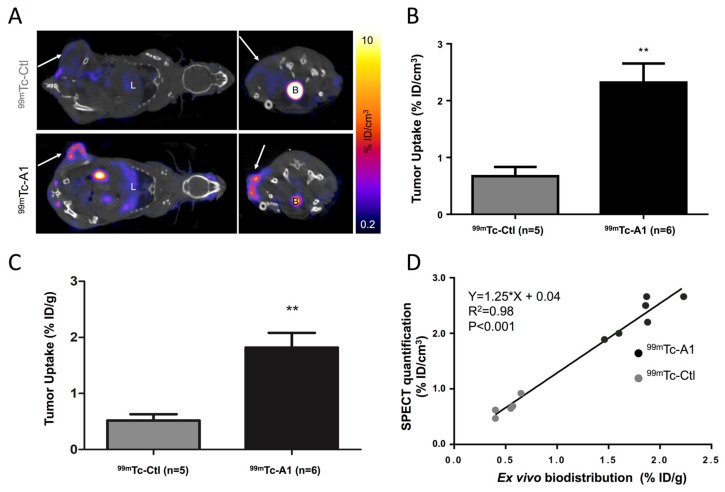
^99m^Tc-A1 binds to AsPC-1 tumor in vivo. (**A**) Representative coronal and transversal views of fused SPECT-CT images of AsPC-1 tumor-bearing mice one hour after IV injection of ^99m^Tc-Ctl (n = 5) or ^99m^Tc-A1 (n = 6). B: bladder and L: liver. Tumor is indicated by the white arrow. (**B**) In vivo quantification of ^99m^Tc-A1 and ^99m^Tc-A1 tumor uptake from SPECT images. (**C**) Ex vivo quantification of ^99m^Tc-A1 tumor uptake from postmortem analysis. (**D**) Correlation between tumor uptake assessed by SPECT and biodistribution. ** *p* < 0.01 vs. ^99m^Tc-Ctl.

Coronal and transversal views of fused Single Photon Emission Computed Tomography (SPECT-CT) images are shown in Figure 3A. ^99m^Tc-A1 uptake in mesothelin-positive AsPC-1 cells was readily identifiable, whereas a weak signal was detected using the irrelevant control sdAb (Figure 3A). This observation was further confirmed by image quantification showing that ^99m^Tc-A1 uptake was 3.5-fold higher than ^99m^Tc-Ctl uptake in AsPC-1 tumor-bearing mice (2.4 ± 0.6 vs. 0.7 ± 0.2% ID/cm^3^, P < 0.01) (Figure 3B). This result was then confirmed by ex vivo gamma-well counting showing that the ^99m^Tc-A1 condition displayed a significant greater uptake (P < 0.01) (Figure 3C). Linear regression analysis confirmed the observations from both in vivo and ex vivo quantifications (Y = 1.25 × X + 0.04, r^2^ = 0.98, P < 0.001) (Figure 3D). Thus, these results validate the use of ^99m^Tc-A1 in assessing in vivo MSLN expression in PDAC.

## 3. Discussion

Despite intense clinical efforts, PDAC remains one of the most aggressive malignancies with a five-year survival rate around 5–7% [3]. The advanced stages of PDAC at the diagnosis and the intrinsic resistance to standard therapies give rise to this dismal prognosis [7]. Until today, most clinical trials have failed to demonstrate significant improvement in patient survival [10]. Identification of targetable molecules for PDAC early diagnosis and treatment thus represents an urgent need. Among the potential targets, current evidence includes mesothelin as a PDAC biomarker. Mesothelin expression is restricted to mesothelial cells (pleura, peritoneum, and pericardium) and its physiological function still remains unknown. Indeed, mesothelin seems to be nonessential in normal tissues [11]. However, mesothelin is present in a wide range of tumors including ovarian and lung cancers (60–65%), and also PDAC-derived tumors (80–85%) [12]. Several studies demonstrated a crucial role of mesothelin in cell survival, proliferation, and resistance to chemotherapy [7,13].

Using TCGA datasets, we observed an overexpression of mesothelin only in PDAC-derived specimens in comparison to nontumoral samples. In agreement with other previous studies, high expression of mesothelin predicted shorter overall survival of PDAC patients [7,14]. The increase of mesothelin expression in the advanced disease stages of PDAC suggests its relevance as a disease progression biomarker. Accumulating evidences suggest its role as a diagnostic marker and a therapeutic target [7]. Several strategies including monoclonal antibodies (mAbs), vaccines, or immunotoxins are currently under clinical evaluation [7]. Importantly, soluble mesothelin assays failed in validating their diagnostic potential for PDAC detection despite overexpression of the membrane-bound form [15,16]. No significant variation of serum mesothelin levels could be evidenced regardless of the different stages of PDAC progression [17]. Nevertheless, identification of patients eligible for such anti-mesothelin therapies still remains a perplexing issue.

Nuclear imaging represents a highly sensitive and noninvasive imaging modality that could address this challenge. Indeed, we previously validated ^99m^Tc-A1, which is a single-domain antibody-derived imaging agent, as an efficient probe in accurately targeting mesothelin-expressing triple-negative breast cancer [9]. Herein, we evaluated the potential of ^99m^Tc-A1 as an imaging probe of mesothelin-expressing PDAC. Our in vivo experiments showed that ^99m^Tc-A1 enabled the noninvasive visualization of AsPC-1-derived tumors by SPECT imaging at early time points. These results reinforce our study on triple-negative breast cancer, despite the lower ^99m^Tc-A1 uptake of PDAC mesothelin-positive tumors [9]. In addition to triple-negative breast cancer and PDAC, ^99m^Tc-A1 imaging represents a relevant option to visualize other aggressive cancers overexpressing mesothelin such as ovarian and lung cancers, for which the imaging modalities are mainly based on the use of conventional antibodies. However, further in vivo competition experiments using unlabeled A1 would confirm the specificity of ^99m^Tc-A1 uptake by PDAC-derived tumors as previously established in breast cancer [9]. To our knowledge, all mesothelin-targeted radiotracers indeed rely on the production of mAbs or single-chain variable fragments (scFv) [18,19,20]. Their hepatic elimination and their slow blood clearance are the main limitations for their use as imaging probes. The smaller size of sdAb-based imaging agents allows: (1) a fast blood clearance and (2) image acquisitions with high target-to-background ratios as early as one hour following administration. Future perspectives of this work include evaluation of the sensitivity of ^99m^Tc-A1 and its ability to phenotype mesothelin-expressing tumors in an orthotopic model of PDAC. These results support further preclinical development of ^99m^Tc-A1 and translation to human applications for cancers that overexpress mesothelin.

Future directions of this work would therefore include clinical translation of ^99m^Tc-A1 for the identification of mesothelin-expressing PDAC that would allow selection of patients who might benefit from mesothelin-targeted therapies that are currently undergoing clinical trials [6].

## 4. Materials and Methods

### 4.1. Patients Online Datasets

Gene expression levels of *mesothelin (MSLN*), *MKI67*, *CCNB1*, and *PCNA* were analyzed and compared in tumor (T) and normal tissue (NT) from PDAC-derived specimens using PAAD (Pancreatic adenocarcinoma), TCGA (The Cancer Genome Atlas), and GTEx datasets (T: n = 179 and NT: n = 171) through the available interface Gene Expression Profiling Interactive Analysis (GEPIA) [13]. The results published here are based upon data generated by the TCGA Research Network [21]. Survival analysis was performed using the Kaplan–Meier (KM) Plotter Database as already described [22,23,24]. The gene expression was extracted from the TCGA database, with at least five-year follow-up data from PDAC patients (n = 177). The data were not adjusted for clinical status and to analyze the prognostic value of *MSLN*, *MKI67*, *CCNB1*, and *PCNA* genes, the KM method was used to estimate survival curves. Hazard ratio (HR) and overall survival were calculated at the best auto-selected cut-off. *p*-values were calculated using the log-rank test to compare survival curves of high and low gene expression groups and a *p*-value below 0.05 was considered to be statistically significant.

### 4.2. Cell Lines Gene Expression Data and Visualization

Preprocessed microarray gene expression data deposited in ArrayExpress (E-MTAB-3610, EMBL-EBI, Cambridgeshire, UK) were downloaded from the Genomics of Drug Sensitivity in Cancer (GDSC) web page [25]. The gene symbols were mapped to Ensembl gene IDs by using the R package biomaRt v.2.40.3 with the human genome version GRCh37.p13. The R package pheatmap v.1.0.12 was used to visualize the gene expression data using hierarchical clustering with Euclidean distance and “complete” agglomeration method.

### 4.3. Cell Lines and Culture Conditions

AsPC-1, SW1990, and MIAPaCa-2 cell lines were maintained at 37 °C and 5% CO_2_ and cultured in DMEM medium (4.5 g/L glucose, 2 mM L-glutamine, 1 mM Sodium Pyruvate) supplemented with 10% fetal bovine serum and 1% penicillin–streptomycin.

### 4.4. Immunoblotting

Total proteins from AsPC-1, SW1990, and MIAPaCa-2 cells were extracted using RIPA buffer (Thermo Fisher Scientific, Illkirch, France). After migration in 12% SDS-polyacrylamide gel, proteins were transferred to nitrocellulose membranes (90 min, 100 V). They were saturated in PBS-Tween 0.1% containing 2% milk at room temperature for 1 h and then probed overnight with the antimesothelin antibody (1/2000, Boster Immunoleader, Pleasanton, CA, USA). Membranes were then stripped during 10 min (0.1% SDS, 1.5% glycin, 1% Tween, pH 2.2) for β-actin detection (1/10 000, Beckton Dickinson, Le Pont de Claix, France).

### 4.5. In Vitro Binding Studies

A1 and irrelevant control single-domain antibody (sdAb) were radiolabeled with technetium-99m (^99m^Tc) using the tricarbonyl method as previously described [9]. For in vitro studies, 200,000 AsPC-1, SW1990, and MIAPaCa-2 cells were incubated with 40 nM of ^99m^Tc-A1 for 1 h at 4 °C. After five washes in cold PBS, the radioactivity was determined using a γ-counter (Wizard², Perkin Elmer, Courtaboeuf, France). Unspecific binding of ^99m^Tc-A1 was determined on MIAPaCa-2 cells and was subtracted from ^99m^Tc-A1 binding on SW1990 and AsPC-1 cells. Results were expressed in counts per minute (CPM).

### 4.6. Tumor Model, SPECT-CT Imaging, and Postmortem Analysis

All animal procedures conformed to French government guidelines (Articles R214-87 to R214-126; European directive 2010/63/UE). They were performed in an approved facility (C385161 0005) under permit APAFIS#3690-2016011916045217 v4 from the French Ministry of Research. Four million AsPC-1 cells were subcutaneously injected into the left flank of five-week-old female Swiss Nude immunodeficient mice (n = 11), in a 2/1 (v/v) PBS/Matrigel (Corning) mix. Tumors were allowed to grow until they reached 200 mm^3^. AsPC-1-tumor-bearing mice were either injected with irrelevant control ^99m^Tc-Ctl (n = 5) or ^99m^Tc-A1 (n = 6). SPECT-CT acquisitions were performed one hour after injection of 42.1 ± 9.0 MBq of ^99m^Tc-sdAbs. ^99m^Tc-Ctl and ^99m^Tc-A1 tumor uptake was expressed in % ID/cm^3^. Two hours after injection and immediately following SPECT-CT image acquisitions, anesthetized mice were euthanized using CO_2_ and tumors were harvested and weighed, and tracer activity was determined with a γ-counter (Wizard^2^, Perkin). Results were corrected for decay, injected dose, and tumor weight, and expressed as % ID/g.

### 4.7. Statistics

Results are expressed as means ± standard deviation and analyzed with GraphPad Prism software (Version 6, software, San Diego, CA, USA). Differences between groups were analyzed using an unpaired Mann–Whitney test for intergroup analysis. Significance of linear correlations was assessed using a Pearson’s test. A *p* value below 0.05 was considered significant (* P < 0.05, ** P < 0.01, *** P < 0.001).

## 5. Conclusions

^99m^Tc-A1 allows imaging of mesothelin-expressing PDAC. Our study represents the first step to using this technology as a companion test to select patients eligible for mesothelin-targeted therapies.

## Supplementary Materials

The following are available online at https://www.mdpi.com/2072-6694/11/10/1531/s1, Figure S1: Markers of proliferation are associated with decreased overall survival in patients with PDAC. Figure S2: Mesothelin expression of MIAPaCa-2, SW1990 and AsPC-1 was assessed by Western Blot.
