# Supplementary material for: 99mTc-A1 as a Novel Imaging Agent Targeting Mesothelin-Expressing Pancreatic Ductal Adenocarcinoma
Source: Cancers (Basel). 2019 Oct 10;11(10):1531. doi: 10.3390/cancers11101531 (PMC6827014; doi:10.3390/cancers11101531)
Supplement: Supplementary file 1 [file cancers-11-01531-s001.pdf]

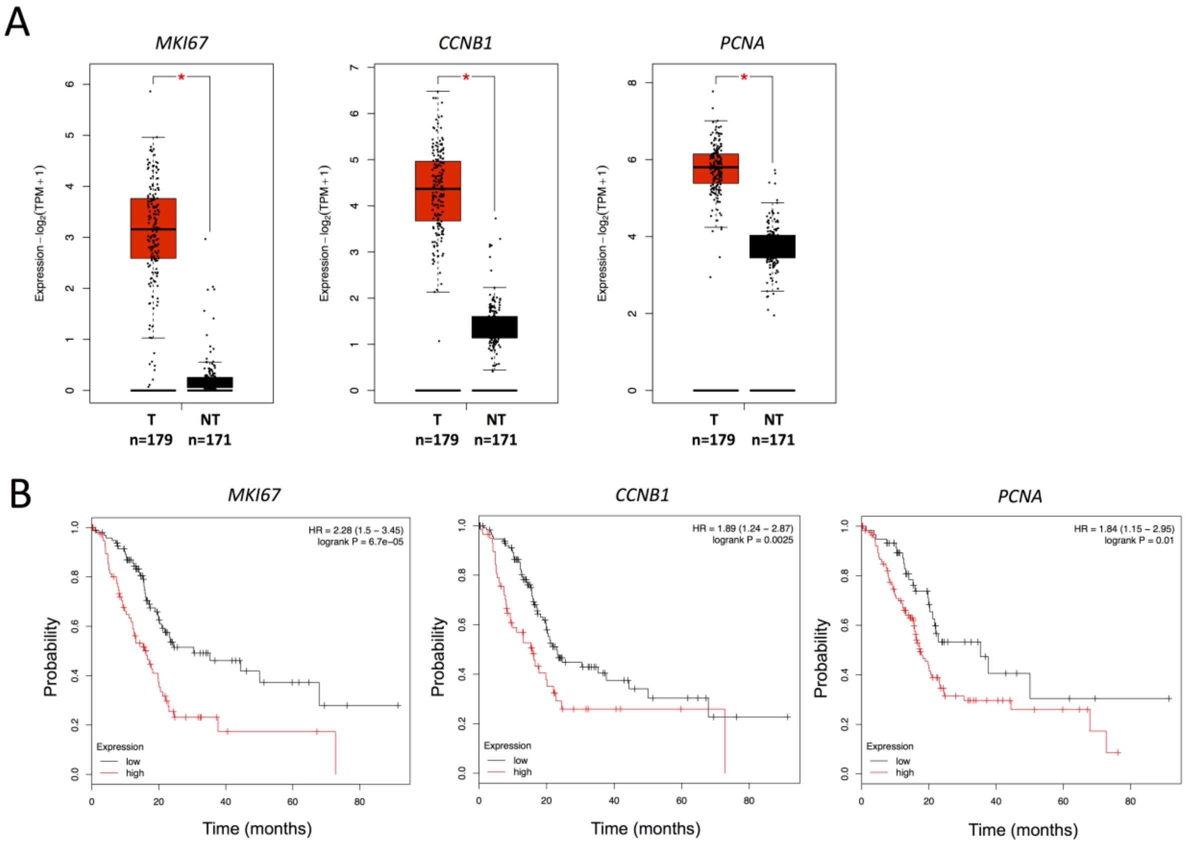

Figure S1. Markers of proliferation are associated with decreased overall survival in patients with PDAC. (**A**) The expression of *MKI67*, *CCNB1* and *PCNA* in tumoral (T) and non-tumoral (NT) pancreatic tissues from TCGA and GTEx datasets. The red and gray boxes represent PDAC and non-tumoral-derived tissues respectively; (T: n = 179 and NT: n = 171). (**B**) KM plots of Overall survival probability (plotted on Y-axis) of PDAC cancer patients is shown (TCGA data, n = 177). Patients have been stratified into high (red lines) or low (black lines) expression-based 'risk-groups' by their gene-expressions of *MKI67*, *CCNB1* and *PCNA*. The patient follow-up is indicated in months on the X-axis. Respective Log-rank test *P*-value and HR are shown and were calculated at the best auto-selected cut-off.

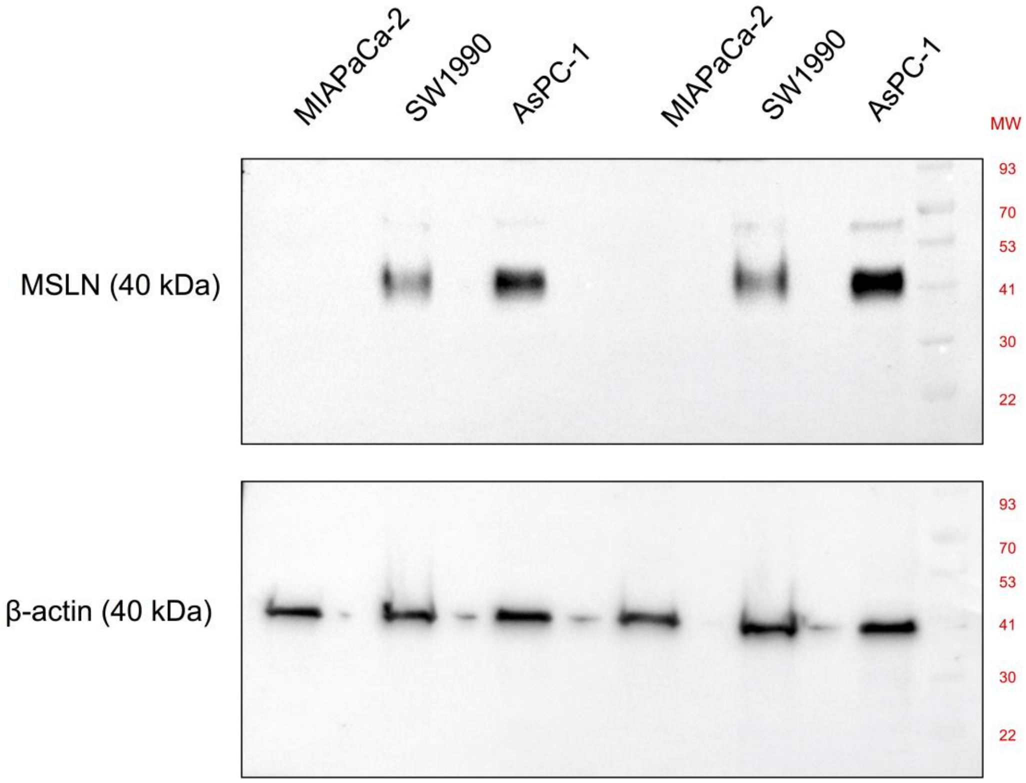

		MIAPaCa-2	SW1990	AsPC-1
Band intensity (grey values)	β-Actin	53296<br>69120	63461<br>87785	69266<br>69929
	Mesothelin	ND	38268<br>56643	71392<br>95039

Figure S2. Mesothelin expression of MIAPaCa-2, SW1990 and AsPC-1 was assessed by Western Blot. MW: Molecular Weight. Whole blots pictures depicting mesothelin and β-actin protein levels (n = 2). Grey values of bands are indicated in the table. ND: Not detected.
